# Trauma center accessibility for road traffic injuries in Hanoi, Vietnam

**DOI:** 10.1186/1752-2897-5-11

**Published:** 2011-09-30

**Authors:** Takashi Nagata, Ayako Takamori, Yoshinari Kimura, Akio Kimura, Makoto Hashizume, Shinji Nakahara

**Affiliations:** 1 Kyushu University Hospital, Department of Emergency and Critical Care Center, Fukuoka, Japan; 2 Kurume University Graduate School of Medicine, Kurume, Japan; 3 Osaka City University, Graduate School of Literature and Human Science, Osaka, Japan; 4 National Center for Global Health and Medicine, Department of emergency medicine and critical care, Tokyo, Japan; 5 St. Marianna University School of Medicine, Department of Preventive Medicine, Kawasaki, Japan

## Abstract

**Background:**

Rapid economic growth in Vietnam over the last decade has led to an increased frequency of road traffic injury (RTI), which now represents one of the leading causes of death in the nation. Various efforts toward injury prevention have not produced a significant decline in the incidence of RTIs. Our study sought to describe the geographic distribution of RTIs in Hanoi, Vietnam and to evaluate the accessibility of trauma centers to those injured in the city.

**Methods:**

We performed a cross-sectional study using Hanoi city police reports from 2006 to describe the epidemiology of RTIs occurring in Hanoi city. Additionally, we identified geographic patterns and determined the direct distance from injury sites to trauma centers by applying geographical information system (GIS) software. Factors associated with the accessibility of trauma centers were evaluated by multivariate regression analysis.

**Results:**

We mapped 1,271 RTIs in Hanoi city. About 40% of RTIs occurred among people 20-29 years of age. Additionally, 63% of RTIs were motorcycle-associated incidents. Two peak times of injury occurrence were observed: 12 am-4 pm and 8 pm-0 am. "Hot spots" of road traffic injuries/fatalities were identified in the city area and on main highways using Kernel density estimation. Interestingly, RTIs occurring along the two north-south main roads were not within easy access of trauma centers. Further, fatal cases, gender and injury mechanism were significantly associated with the distance between injury location and trauma centers.

**Conclusions:**

Geographical patterns of RTIs in Hanoi city differed by gender, time, and injury mechanism; such information may be useful for injury prevention. Specifically, RTIs occurring along the two north-south main roads have lower accessibility to trauma centers, thus an emergency medical service system should be established.

## Introduction

In Vietnam, road traffic injuries (RTIs) are becoming a major public health issue [1]. These injuries occur more frequently due to rapid economic growth and motorization in recent years; indeed, the number of road traffic fatalities nationally rose from 4,907 in 1994 to 11,534 in 2005. RTI are the leading cause of death in the 15 - 59 years age group, while RTI is the second in men and the fifth in women most frequent causes of death among the total population [2]. Economic loss also results from RTIs, and is currently estimated at about 855 million US dollars (or 2.45% of Gross Domestic Product) per year [1,3].

Policy changes are needed to mitigate this major public health issue. For example, injury prevention programs are effective in reducing RTIs: the helmet law enacted by the Vietnamese government in December 2007 increased the incidence of helmet use among motorcyclists to around 85% and substantially decreased motorcycle-related head injuries (-16%) and fatalities (-18%) [4,5]. Additional prevention programs may result in declines in RTIs. Alternatively, an emergency medical service (EMS) can be effective at reducing losses associated with RTI, by streamlining the chain of survival [6]. While Vietnam has some EMSs, they are not structured, insurance issues are often undetermined, and the real performance of the EMS remains unclear. Effective EMS systems depend upon the prompt transportation of injured people from the scene to the trauma center. Transportation time is directly influenced by the location of the RTI occurrence, of the ambulance dispatch center, and of the trauma center. Therefore, examining the geographical distribution of RTI occurrence and the distance between injury scenes and trauma centers can provide useful information in planning EMS systems, particularly new ambulance dispatch centers or trauma centers.

A useful tool for investigating such geographical factors is the geographical information system (GIS). GIS is widely applied to various areas of scientific research, including RTIs [7,8], and offers two advantages: visual results and subsequent application of spatial analysis. For our study, GIS was used to determine accessibility to trauma centers from injury scenes. Specifically, we aimed to describe the geographical distribution of RTIs and evaluate the accessibility to trauma centers for those injured in Hanoi, Vietnam.

## Methods

We performed a cross-sectional study using data from the Hanoi city police agency. Hanoi is the capital of Vietnam, with an estimated population of 6,232,940 people in 2008. The city has experienced rapid economic growth since the 1990s, with a corresponding increase in motor vehicle use. In 2004, with the support of the Japanese government, the Hanoi city police and city traffic agency established a database for RTI. Following a road traffic incident in Hanoi--for example, a motorcycle versus motorcycle collision--if police were contacted, the incident was investigated and registered in the database. Major injury or fatality cases were often reported to the police. Road traffic fatality was defined as a death occurring at the scene of a road traffic incident and road traffic injury was defined as severe cases caused by road traffic incidents in this database. In 2006, database entries were not subjected to specific inclusion criteria: however, severe and fatal cases were entered in this database.

Anonymous, registered cases of road traffic injuries--including fatalities--occurring in Hanoi between January 1^st^, 2006 and December 31^st^, 2006 were included in our study. Data retrieved for each case included age, gender, time of injury, injury mechanism (motorcycle versus motorcycle, motorcycle versus car, motorcycle versus bicycle, motorcycle versus pedestrian, motorcycle versus train, car versus car, car versus bicycle, car versus pedestrian, car versus train, etc), and injury location.

We evaluated the basic characteristics for RTIs, and then mapped the cases to Hanoi using GIS software ArcMap 9.2 (ESRI Co Ltd) and address matching. Kernel density estimation, a non-parametric approach to estimating a probability density function for random variables, was applied to determine the incidence of road traffic fatalities/injuries. In GIS, Kernel density estimation is used to calculate the magnitude per unit area using a Kernel function to fit a smoothly tapered surface to each point. In this analysis, the radius, or band-width, was set to 1,000 meters. Viet Duc Hospital, Bach Mai Hospital, and Saint Paul Hospital were active trauma centers in Hanoi in 2006. These hospitals are located near one another at the center of Hanoi. The direct distance between site of road traffic injury and each of these three hospitals was measured to determine accessibility of RTIs to trauma centers.

Multivariate linear regression analysis was used to investigate factors associated with the distance between injury location and trauma centers. The outcome variable of the regression model was the direct distance (km) between the site of road traffic injury and Viet Duc Hospital, which has the largest trauma center in Hanoi, Vietnam. Road traffic fatality or injury, age groups (20-29 years of age or other), gender (male or female), time (morning: 0 am-11 am, afternoon: 11 am-6 pm, night: 6 pm-0 am), day (weekday: Monday-Friday, weekend: Saturday and Sunday) and injury mechanism (motorcycle versus motorcycle, motorcycle versus car, bicycle-associated, pedestrian-associated and others), were used as explanatory variables. SAS 9.2 was used in data analysis. *p*-values less than 0.05 were accepted as statistically significant. The study was approved by the Institutional Review Board at Kurume University, Japan.

## Results

During the study period, 1,490 road traffic injuries were registered. Among these, data for injury location/site of road traffic incident were documented in 1,271. Thus, 209 cases either lacked data for injury location or the address could not be located on the map of Hanoi.

Demographic characteristics of RTIs in Hanoi in 2006 are shown in Table 1. Cases tended to be 20-29 years of age and male. Additionally, more than 63% of RTIs/fatalities were motorcycle versus motorcycle/car.

**Table 1:** Basic characteristics of road traffic injuries in Hanoi, 2006

Variable	N	Mean (SD) or Percentage
Distance (km)	1271	6.781 (5.316)
Fatality or Injury		
Fatality	424	41%
Injury	610	59%
Total	1034	100%
Gender		
Male	818	79%
Female	216	21%
Total	1034	100%
Age groups		
20 - 29 years	423	46%
Other	504	54%
Total	927	100%
Time		
Morning (0 am - 11 am)	382	30%
Afternoon (11 am - 6 pm)	383	30%
Night (6 pm - 0 am)	496	40%
Total	1261	100%
Day		
Weekday	410	32%
Weekend	861	68%
Total	1271	100%
Injury Mechanism		
Motorcycle vs. motorcycle	347	28%
Motorcycle vs. car	427	35%
Pedestrian-associated	157	13%
Bicycle-associated	100	8%
Others	193	16%
Total	1224	100%

SD: Standard Deviation

**Figure 1:**
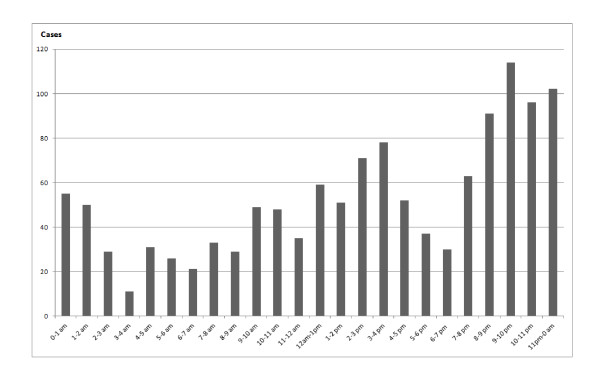
**Time distribution of road traffic injuries occurring in Hanoi, Vietnam**.

**Figure 2:**
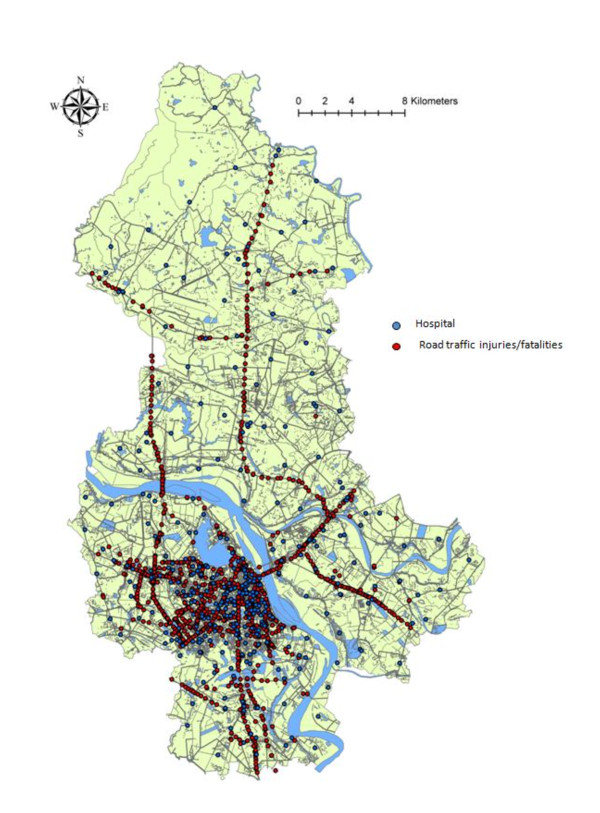
GIS-based map of road traffic injuries (red dots) and hospitals (blue dots) in Hanoi, Vietnam

**Figure 3:**
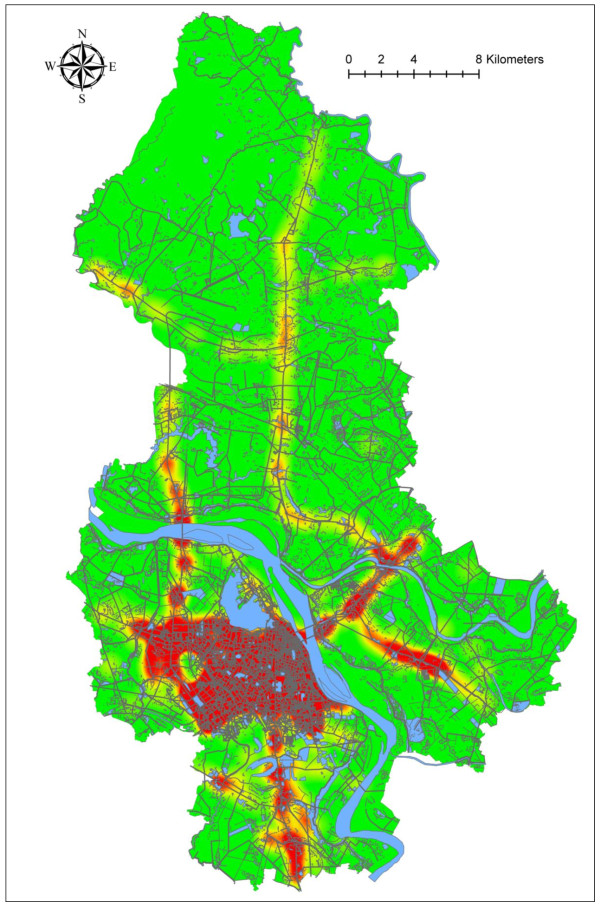
**Kernel density estimation of the occurrence of road traffic injuries in Hanoi, Vietnam**.

Interestingly, RTIs were more likely to occur during two peak times of day: between 12 am and 4 pm and between 8 pm and 0 am (Figure 1). Figure 2 shows the geographical distribution of RTIs (red dot) and hospital/health care centers (blue dot) in Hanoi, while Figure 3 shows the Kernel density of RTIs (band-width of 1,000 meters). RTIs were clustered in "hot zones" in the central city area and along the two north-south main roads (highways, Routes 3 and 8). The hospitals were also clustered in the center of the city, including three major trauma centers (Viet Duc Hospital, Bach Mai Hospital, and Saint Paul Hospital). The median direct distances between injury sites and the three trauma centers were 5.65 (3.19 - 8.64) km for Viet Duc Hospital, 5.31 (2.89 - 8.54) km for Bach Mai Hospital, and 5.11 (3.11 - 8.72) km for Saint Paul Hospital.

The estimations of Pearson's correlation regarding mutual relationships of the explanatory variables were less than 0.125, and the issue of multi-colinearity could be omitted.

The result of a multivariate linear regression analysis indicated that the direct distance from the injury sites to Viet Duc Hospital were significantly associated with fatality, gender and mechanism of injury (Table 2). Compared with RTI, road traffic fatalities occurred 1.538 km farther from Viet Duc hospital significantly (*p*-value < 0.0001). Compared with females, male RTIs occurred 1.082 km farther from Viet Duc hospital significantly (*p*-value = 0.019). Finally, compared with motorcycle versus motorcycle incidents, motorcycle versus car incidents occurred 2.715 km farther from Viet Duc Hospital significantly (*p*-value < 0.001). Similar results were observed for Bach Mai and Saint Paul hospitals (not shown).

**Table 2:** The adjusted effects of fatalities, gender, age group, time, day, and injury mechanism on trauma center access (Viet Duc Hospital) for road traffic injury by a multiple regression model

Variable	Coefficient	SE	*p- *value
Fatality or Injury^1^			
Fatality	1.538	0.378	<0.0001
Gender^2^			
Male	1.082	0.458	0.019
Age groups^3^			
20 - 29 years	0.394	0.373	0.292
Time^4^			
Morning (0 am - 11 am)	-0.618	0.451	0.171
Afternoon (11 am - 6 pm)	-0.024	0.445	0.957
Day^5^			
Weekday	0.560	0.395	0.156
Injury Mechanism^6^			
Motorcycle vs. car	2.715	0.470	<0.0001
Bicycle-associated	1.311	0.761	0.085
Pedestrian-associated	0.594	0.638	0.352
Others	0.722	0.571	0.207

^1 ^Compared with injury (reference category)

^2 ^Compared with female (reference category)

^3 ^Compared with other ages (reference category)

^4 ^Compared with night (reference category)

^5 ^Compared with weekend (reference category)

^6 ^Compared with motorcycle vs. motorcycle (reference category)

## Discussion

Our study determined the geographical distribution of RTIs in Hanoi in 2006. RTIs were clustered in the center of the city and along the two major north-south roads (Routes 3 and 8). As shown in Table 2, the multiple regression analysis showed that the direct distance from the injury sites to the major trauma center (Viet Duc Hospital) was statistically longer with road traffic fatality, male or motorcycle vs. car incidents. This finding likely reflects a predominance of cars on major roads outside the city area, such as Routes 3 and 8, high risk-taking behavior in males (high speed and alcohol driving), and lack of a prehospital care system [9]. Also, male motorcyclists tend to drive outside downtown while female motorcyclists drive inside downtown.

In Vietnam, RTI is one of the leading causes of morbidity and mortality. Correspondingly, great effort has been made to prevent these injuries, with changes in traffic regulations and helmet law endorsement, and to better treat them through improved trauma center care and EMS [4,5,9-12]. We recommend additional measures to reduce the effects of road traffic injuries in Hanoi city. EMS dispatch centers along Routes 3 and 8 or pre-hospital transport by commercial vehicles would improve accessibility to care following a road traffic incident in these "hot zones" [13,14]. Indeed, new trauma centers along Routes 3 and 8 would be more effective at reducing RTIs; however, limited finances and infrastructure conditions may prohibit such a measure.

There are several limitations to this study. First, the data used in this study were not linked with hospital records, and clinical outcomes could not be confirmed. Second, only severe/fatal road traffic incidents were included in the registry, thus under-reporting of incidents was possible [15]. However, the occurrence of a number of RTIs far from trauma centers was not influenced by potential under-reporting. While data collection by the Hanoi city police agency was supported by international cooperation from Japan at an early stage of the project, data quality might be insufficient [16]. Third, GIS data were not fully available in Hanoi in 2006, and further geo-statistical analysis could not be performed in this study. Fourth, to accurately evaluate accessibility of trauma centers, travel time and the actual distance traveled by a vehicle--rather than shortest distance on a map--should be assessed. This approach would be helpful in determining whether the EMS system should be "scoop and run" or "stay and play" [17]. However, an important limitation of our study was that we did not have access to data regarding travel time or actual distance traveled. Therefore our analysis did not include these variables.

GIS is widely used in emergency medicine research [18-20]. In a retrospective study, Peleg et al. used GIS to simulate ambulance access to trauma care in two regions of Israel. They found that, in these two regions, 34% and 62% of calls could be responded to within 8 minutes [21]. A similar approach could not be used in our study because of limited information; however, visualization of the sites where RTIs occur can aid in determining the allocation of EMS dispatch centers or trauma centers.

Despite these limitations, our study was one of the first to apply GIS for RTI research in a low-income country. Our results indicate that pre-hospital care should be implemented to reduce the effects of road traffic incidents in Hanoi. For example, building new EMS dispatch centers along Routes 3 and 8 or establishing alternative pre-hospital transportation by commercial vehicles or private ambulance would provide easier access to trauma centers in the city. We also would like to apply GIS to improve trauma management in industrialized countries such as Japan in the future. We believe that research and infrastructure to link GIS systems with hospital, EMS, or police data are needed.

In conclusion, this study has identified the geographical distribution of road traffic injuries and accessibility to trauma care in Hanoi, Vietnam. The observations provide clues for injury prevention and safety promotion by a multi-disciplinary approach. In particular, we recommend developing a more efficient EMS system in Hanoi. Finally, we note that GIS may help to collect, analyze, and integrate geographical and environmental data on road traffic injuries.

## Competing interests

The authors declare that they have no competing interests.

## Authors' contributions

TN, YK and AK originated and designed the study. TN, AT, MH, and SH interpreted the results and commented on drafts of the articles. AT and YK carried out geographical and statistical analysis. All the authors approved the final version, and will take the responsibility for the content of the paper.
